# *Coreopsistinctoria* Nutt. Alleviates Intestinal Barrier Damage in Slow Transit Constipation Through the PI3K/AKT Pathway

**DOI:** 10.3390/cimb48050510

**Published:** 2026-05-14

**Authors:** Guliziremu Ainiwaer, Xiaoxuan Zhang, Mukatansi Tayier, Xin Luo

**Affiliations:** College of Pharmacy, Xinjiang Medical University, Urumqi 830011, Chinam18196001753@163.com (X.Z.); 17590821975@163.com (M.T.)

**Keywords:** slow transit constipation, *Coreopsis tinctoria* Nutt., intestinal barrier dysfunction, network pharmacology

## Abstract

**Background**: The development of Slow Transit Constipation (STC) is associated with intestinal barrier damage. *Coreopsis tinctoria* Nutt. (CT) is effective in treating STC, but the mechanisms are unclear. **Methods**: We investigated three CT extracts—traditional aqueous extract, and an aqueous extract from supercritical fluid extraction, with or without lipophilic components—on intestinal transit in a loperamide-induced STC rat model. The potential therapeutic targets of CT for STC were initially predicted using an integrated approach of network pharmacology and molecular docking. The therapeutic effect of CT was evaluated in a STC rat model by assessing defecation parameters (fecal count, water content, intestinal transit), colon histology (H&E and AB-PAS staining), inflammatory markers (ELISA), and target protein expression (Western blotting and immunohistochemistry). In parallel, an LPS-induced IEC-6 cell injury model was used to investigate intestinal barrier protection, analyzing cell viability (CCK-8), apoptosis (flow cytometry and Western blotting), migration (scratch assay), and protein expression (Western blotting). **Results**: Docking and enrichment analysis highlighted hub targets (TNF, AKT1, Caspase3, STAT3, and BCL-2) and the PI3K/AKT pathway. In vivo, CT treatment improved defecation function, reduced colonic damage, and decreased markers of inflammation and apoptosis in STC rats. It also up-regulated ZO-1 and Occludin, lowered serum markers of intestinal permeability D-lactate (D-LA) and Diamine oxidase (DAO), and restored intestinal barrier function. Furthermore, CT reduced Caspase3 expression and increased the expression of proteins such as BCL-2, PI3K, and P-AKT/AKT. These findings were further supported by in vitro experiments. **Conclusions**: CT improves STC and its associated intestinal barrier damage by activating the PI3K/AKT pathway and suppressing inflammation and apoptosis, among which the aqueous extract from supercritical fluid extraction combined with the lipophilic fraction exhibits the best efficacy.

## 1. Introduction

As dietary imbalances intensify, living rhythms accelerate, and psychological stress increases, constipation has become one of the most prevalent gastrointestinal disorders worldwide [1,2], affecting approximately 10.1% to 15.3% of the global population [3]. Among its subtypes, slow transit constipation (STC) represents the primary clinical form, accounting for 46% to 74% of chronic constipation cases [4]. Characterized by reduced colonic motility and prolonged intestinal transit time, STC commonly leads to symptoms such as difficult defecation, abdominal distension, and pain [5]. In severe cases, it may trigger acute intestinal obstruction, intestinal necrosis, or perforation [6]. This damage may also raise the risk of cardiovascular and cerebrovascular problems, significantly impairing patients’ quality of life [7,8]. Therefore, elucidating the pathogenesis of STC and exploring more effective therapeutic strategies carry substantial clinical importance.

Evidence increasingly points to the intestinal barrier as a key factor in how STC develops [9]. Tight junction proteins (ZO-1 and Occludin) together with an intact epithelial cell layer form this barrier. Intestinal inflammation and abnormal apoptosis are key factors inducing this dysfunction [10,11]. Different types of proinflammatory cytokines can interfere with the structural integrity and physiological function of tight junction proteins through differentially regulated pathways, ultimately inducing functional damage to the intestinal mucosal barrier [12,13]. Furthermore, studies have revealed significantly increased apoptosis in colonic epithelial cells during constipation [14]. In practice, this shows up as more Caspase3 and less BCL-2. The resulting imbalance only makes the barrier damage worse. Thus, disruption of inflammatory cytokine secretion and abnormal apoptosis-induced damage to the intestinal mucosal barrier represent one of the pathogenesis mechanisms of STC. This suggests that regulating intestinal inflammatory balance and apoptosis to repair intestinal epithelial barrier function may offer a novel therapeutic strategy for STC.

Traditional Chinese Medicine (TCM) has been widely used in the treatment of gastrointestinal disorders [15] and has demonstrated unique advantages in managing constipation [16]. However, its multi-component, multi-target mechanisms of action have not been fully elucidated, which to some extent limits its clinical translation and international promotion [17]. Network pharmacology, an emerging research method that integrates systems biology and multi-omics technologies, offers an efficient approach to clarify the holistic regulatory mechanisms of TCM by constructing compound-target-pathway interaction networks.

*Coreopsis tinctoria* Nutt. (CT) is an annual herb belonging to the genus Coreopsis of the family Asteraceae [18]. It is a valuable plant used for both medicinal and culinary purposes, primarily distributed in northwestern China and widely cultivated throughout the country. It has recently been shown, through studies on its chemical composition and pharmacology, that CT is rich in amino acids, polysaccharides, polyphenols, organic acids, flavonoids, volatile oils, steroids, and coumarins [19]. It has been demonstrated to possess antioxidant, anti-inflammatory, immunomodulatory, and antibacterial properties [18,20], and has been shown to effectively lower blood lipid levels [21], treat chronic enteritis [22], and treat hyperuricemia [23]. Previous research by our group has found that one of the primary active compounds in CT, flavanomarein, improves intestinal function by increasing the abundance of dominant beneficial gut bacteria such as *Bacteroidotaenae*, *Muribaculaceae*, and the *Eubacterium coprostanoligenes group*, while simultaneously reducing serum levels of IL-6 and TNF-α and increasing IL-10 levels [24]. Research by Shi et al. has shown that [22] okanin, another major bioactive component of CT, can restore both the barrier function of colonic epithelial cells and tight junctions in intestinal epithelial cells—through regulation of tight junction proteins, specifically occludin, claudin-3, and ZO-1.

For years, our team has focused on evaluating the function and developing the bioactive compounds found in CT extracts. Previously, we have established a stable process for the extraction and preparation of the CT employed in this study, and identified flavonoid compounds such as marigoldin, chrysanthemum chalcone, flavanomarein, and tagetes 7-O-glucoside using HPLC [25]. Using GC-MS, we identified 22 components in the essential oil of Kunlun snow chrysanthemum, with the main constituents being linalool (63.23%), 3-carene (7.05%), and β-pinen (5.23%) [26]. These findings provide a reliable basis for the subsequent establishment of quality standards and research into the pharmacologically active components.

**Figure 1:**
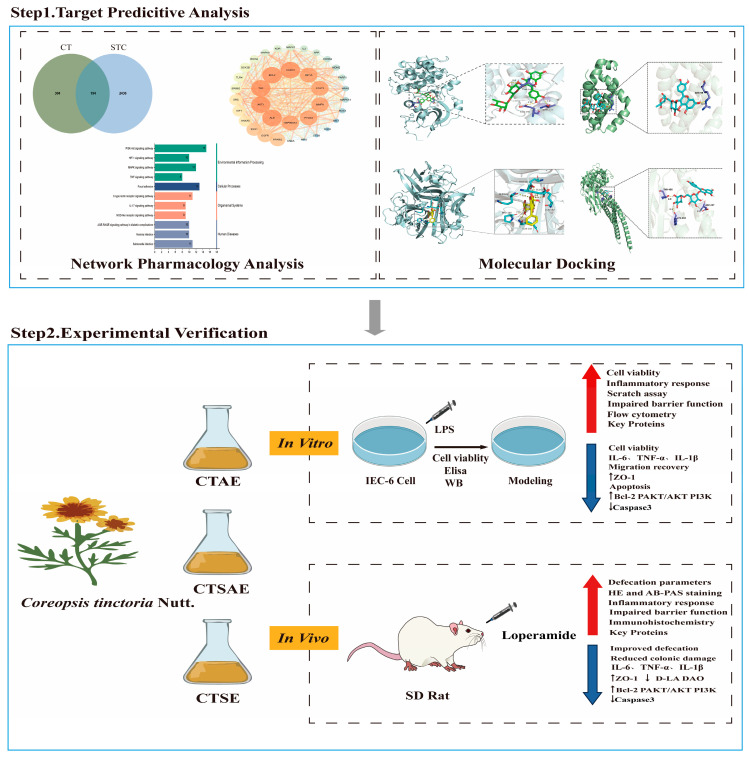
The flowchart reflects our study design. Upward arrows (↑) indicate upregulation of protein expression or increase of the indicator after CTSE treatment; downward arrows (↓) indicate downregulation of protein expression or decrease of the indicator.

Conventional aqueous extraction only recovers water-soluble components such as flavonoids, whereas lipophilic active ingredients like volatile and fatty oils are largely lost during decoction. Unlike traditional extraction methods, supercritical CO_2_ extraction technology offers low extraction temperatures, leaves no toxic residues, and provides selective separation capabilities, making it a highly mature and efficient method for extracting essential oils and fatty oils [19]. Research indicates that lipophilic components, including volatile and fatty oils, have potential effects in promoting gastrointestinal motility and lubricating the intestines. A study on Jichuan Decoction for STC reported that the combination of aqueous extract with volatile oils yielded significantly better efficacy than the aqueous extract alone [27], suggesting the limitations of using a single aqueous extract. Accordingly, we hypothesize that the combination of aqueous extract with lipophilic components (volatile and fatty oils) may exert synergistic effects, resulting in superior efficacy over individual extracts. To test this, we employed supercritical CO_2_ extraction as a preparation strategy to systematically evaluate the therapeutic effects of the CT aqueous extract (CTAE), the CT aqueous extract obtained after supercritical CO_2_ extraction (CTSAE), and an innovative formulation (CTSE) composed of the volatile oil–fatty oil fraction from supercritical CO_2_ extraction combined with the corresponding post-supercritical aqueous extract on STC, thereby providing experimental evidence for developing novel STC therapies based on intestinal barrier repair. The flow diagram of the present study is presented in Figure 1.

## 2. Materials and Methods

### 2.1. Preparation of CT Extracts

CTAE (NO.241024), CTSAE (NO.241024), and CTSE (NO.241018) were provided by China Pharmaceutical University (Nanjing, China). The chemical composition profiles of the extract batches used in this study were consistent with those reported in the literature cited in the Introduction. As previously reported, supercritical CO_2_ extraction primarily removes lipophilic components such as essential oils and fatty acids [19]. We used HPLC to assess the composition of marker compounds in the water extract, using marein and okanin as reference markers [21,22]. Representative chromatograms with preliminary annotations are shown in Supplementary Figure S1. The preparation methods for the various extracts of CT are described below.

#### 2.1.1. Preparation of CTAE

A total of 400 g of CT raw material was subjected to heat-reflux extraction with distilled water at a material-to-solvent ratio of 1:3. The extraction was performed in a water bath at 70 °C for 3 h. After filtration, the extraction step was repeated twice under the same conditions. The three filtrates were pooled and concentrated using a rotary evaporator to obtain a thick paste.

#### 2.1.2. Preparation of CTSAE

A total of 400 g of CT raw material that had previously undergone supercritical-fluid extraction (Batch No.: 20241018) was placed in a 10,000 mL three-neck flask. Ten volumes of water were added, and the mixture was soaked for 30 min. Extraction was then carried out at 80 °C for 2 h using a heating mantle, followed by filtration through a 200-mesh gauze to collect the filtrate. The residue was re-extracted with eight volumes of water under the same conditions (80 °C, 2 h) and filtered. The two filtrates were combined and concentrated under reduced pressure (56 °C) to a viscous liquid (<200 mL), yielding the supercritical-aqueous co-extract concentrate (approx. 2 g crude drug per mL).

#### 2.1.3. Preparation of CTSE

A total of 7.7 g of the supercritical-fluid extract of CT (equivalent to 400 g of raw material) was weighed into a 100 mL beaker. Then, 4 mL of absolute ethanol was added, and the mixture was stirred at 500 rpm for 20 min at room temperature to achieve complete dissolution. Subsequently, 2 g of Tween-80 was added and homogenized. Finally, the supercritical-aqueous co-extract concentrate (equivalent to 400 g of raw material) was incorporated, and the mixture was stirred until uniform. The preparation was diluted to a final volume of 200 mL (solid content approx. 600 mg mL^−1^) and stored at 4 °C.

### 2.2. Reagents

Loperamide (QCJ3886) and Shou Hui Tong Bian Capsules (20241203) were sourced from Xi’an Janssen Pharmaceutical Ltd. (Xi’an, China) and Lunan Houpu Pharmaceutical Co., Ltd. (Linyi, Shandong, China), respectively. All drugs were dissolved in normal saline (0.9%) before being administered to the rats.

### 2.3. Network Pharmacology Profiling

Based on our previous research on the isolation of total flavonoid chemical components and studies related to intestinal absorption, the active constituents of CT were selected. The compound names and molecular structures were confirmed using the PubChem database (https://pubchem.ncbi.nlm.nih.gov/, accessed on 25 March 2024). Potential targets of the active components were predicted with the assistance of the Swiss Target Prediction (http://www.swisstargetprediction.ch/, accessed on 25 March 2024) and PharmMapper databases (http://www.lilab-ecust.cn/pharmmapper/, accessed on 25 March 2024), and all target names were validated via the UniProt database (https://www.uniprot.org/, accessed on 25 March 2024). Using slow transit constipation as the keyword, relevant genes were searched, downloaded, and integrated from the GeneCards (https://www.genecards.org/, accessed on 26 March 2024) and OMIM databases (https://omim.org/, accessed on 26 March 2024). The Venny 2.1 online tool (https://bioinfogp.cnb.csic.es/tools/venny/index.html, accessed on 26 March 2024) was employed to generate a Venn diagram to obtain intersecting genes between the two sets. Using Cytoscape 3.9.1 software, we constructed a network of CT’s active components and analyzed its topological parameters with the software’s built-in network analysis tools. Degree values were then used to screen for core targets. A Protein–Protein Interaction (PPI) network of therapeutic targets was built via the STRING database (https://string-db.org/, accessed on 28 March 2024). Finally, GO and KEGG pathway enrichment analyses were performed using the DAVID 6.8 database (https://davidbioinformatics.nih.gov/, accessed on 1 April 2024).

### 2.4. Molecular Docking

PubChem provided the 3D structures of flavanomarein, rutin, and baicalin. The PDB database was the source for the PDB structures of AKT (PDB ID:4EKL) [28], STAT3 (PDB ID:6NUQ) [29], TNF-α (PDB ID:1TNF) [30], Caspase3 (PDB ID:1NME) [31], and BCL-2 (PDB ID:4AQ3) [32]. The structures were converted into the mol2 format using Chemdraw 3D, and both formats were imported into AutoDockVina1.2.3 for binding energy calculation. Representative docking poses with relatively better binding affinities were selected for visualization. In addition, the root mean square deviation (RMSD) values were calculated by means of PyMOL2.6.0, and an RMSD value below 2 Å was considered to indicate successful methodology validation [33].

### 2.5. Rat Model of STC Induced by LOP

Fifty-four female SD rats (weight 200 ± 20 g) were provided by the Experimental Animal Center of Xinjiang Medical University (SCXK (Xin) 2023-0001) and housed in an SPF-grade animal facility with free access to sterilized feed and water. The rats were randomly divided into a control group (9 rats) and a model group (45 rats). Each group was administered pure water (10 mL/kg) or loperamide (LOP, 10 mg/kg) via gavage daily for 14 consecutive days, with a gavage volume of 10 mL/kg for both groups. During the modeling period, body weight, food intake, wet weight of feces, and water content were measured weekly; on the final day of the modeling period, 3 rats were randomly selected from each group to evaluate the effectiveness of the constipation model by measuring intestinal transit rate.

After successful model establishment, the remaining 42 model rats were randomly divided into 7 groups (6 rats per group), and a control group (6 rats) was established separately. Based on the results of the preliminary experiment and clinical dose equivalence calculations, the treatment groups were as follows: low-dose CTSE group (CTSE-L, 3.58 mg/kg), medium-dose CTSE group (CTSE-M, 7.16 mg/kg), high-dose CTSE group (CTSE-H, 14.32 mg/kg), positive control Shou Hui Tong Bian group (SHTB, 189 mg/kg), CTAE group (CTAE, 3.48 mg/kg), CTSAE group (CTSAE, 3.48 mg/kg), as well as the model group (MOD) and the normal group (CON). Each group received a single daily oral gavage (volume 10 mL/kg) for 14 consecutive days; the control and model groups received an equal volume of purified water. After the administration period, the rats were anesthetized. Blood was immediately collected for serum biochemical analysis. Colon tissues were harvested for histopathological examination and immunohistochemistry (IHC) analysis. The remaining colon tissues were stored at −80 °C for Western blotting experiments.

### 2.6. Cell Culture and Treatments

IEC-6 cells (iCell-r016) were purchased from Saibai Kang Biotechnology Co., Ltd. (Shanghai, China), cultured in DMEM (6124300) supplemented with 10% fetal bovine serum (A2799564P) sourced from Gibco (Waltham, MA, USA), and maintained at 37 °C under an atmosphere of 50 mL/L CO_2_. LPS (L2880, Sigma-Aldrich, Saint Louis, MO, USA) stimulates macrophages and neutrophils to release inflammatory mediators, damaging intestinal epithelial tight junctions, inducing apoptosis, increasing intestinal permeability, and ultimately triggering inflammation that leads to barrier dysfunction. Based on this evidence, LPS was selected as the inducer to establish an in vitro model of inflammatory injury mimicking intestinal barrier dysfunction [11,34,35]. Dexamethasone (DEX), a well-characterized synthetic glucocorticoid known to attenuate systemic inflammation and preserve intestinal barrier integrity, was used as the positive control in this study.

The IEC-6 cells were divided into the following groups: control group (CON), model group (MOD), CTAE group (CTAE), low-dose CTSAE group (CTSAE-L), medium-dose CTSAE group (CTSAE-M), high-dose CTSAE group (CTSAE-H), low-dose CTSE group (CTSE-L), medium-dose CTSE group (CTSE-M), high-dose CTSE group (CTSE-H), and positive control dexamethasone group (DEX). After 24 h of treatment, relevant indicators of IEC-6 cells were measured.

### 2.7. Cell Viability Assay

The CCK-8 assay kit (AC11L054, Life-iLab, Shanghai, China) was employed for cell viability assessment. IEC-6 cells were seeded in 96-well plates at a density of 1.0 × 10^4^ cells/well. After adhesion, the culture medium was replaced with fresh medium supplemented with the test compounds across a range of concentrations (0, 6.25, 12.5, 25, 50, 100, 200, and 400 μg/mL), and incubation was carried out for 24 h. Then, 100 μL of basal medium containing 10% CCK-8 was added per well. The plates were maintained at 37 °C for 2 h, and absorbance readings at 450 nm were taken using a microplate reader (Tecan, fusion-fx6 edge v.070, Grödig, Austria).

### 2.8. Cell Migration Assay

IEC-6 cells were plated in 6-well plates at a density of 3.5 × 10^5^ cells/well. After attachment, serum starvation was carried out using serum-free DMEM for 6 h. A linear scratch was introduced with a sterile 200 µL pipette tip. The cells were subsequently exposed to graded concentrations of each drug for 24 h. Images of the wound were captured immediately after scratching (0 h) and 24 h following treatment; both time-point images were obtained with a fluorescence microscope (Leica, DMi8, Wetzlar, Germany).

### 2.9. Apoptosis Analysis

Apoptosis was evaluated using the Annexin V/PE dual staining kit (559763, BD Biosciences, San Jose, CA, USA). Following treatment with the three CT extracts for 24 h, cells were collected and washed. The cells were then resuspended in 500 µL binding buffer and combined with 5 µL Annexin V plus 5 µL PE. The resulting solution was left to incubate at room temperature in the dark for 15 min, after which apoptotic rates were quantified via flow cytometry (Beckman, Dxflex, Indianapolis, IN, USA).

### 2.10. Twenty-Four-Hour Defecation and Fecal Water Content

Following treatment, rats were housed in metabolic cages for 24 h for fecal collection, with fecal number, weight measurements, and food intake assessed. Samples were then oven-dried at 60 °C for 12 h. The formula denoted below was utilized to measure the fecal water content: Fecal moisture content (%) = (fresh weight of feces − dry stool weight)/fresh weight of feces × 100%.

### 2.11. Measurement of Intestinal Propulsion Function

After the final drug administration, rats were fasted for 12 h. On the following day, each animal was orally administered 2 mL of India ink (I774775, Aladdin, Shanghai, China). Thirty minutes later, the animals were euthanized under anesthesia. The intestinal tract was excised, and the total length from the pylorus to the ileocecal junction was measured, as well as the migration distance of the farthest point of India ink. Intestinal propulsion rate was calculated as follows: Intestinal propulsion rate (%) = (Migration distance of India ink/Total intestinal length) × 100%.

### 2.12. Detection of Biochemical Indicators

The levels of IL-1β (ER1094), IL-6 (ER0042), and TNF-α (ER1393) in cell supernatants and rat serum were measured according to the ELISA kit instructions (Wuhan Fine Biotech Co., Ltd., Wuhan, China). The levels of D-LA (YX-E20582) and DAO (YX-E20552) in rat serum were determined using enzyme-linked ELISA (Shanghai Youxuan Biotechnology Co., Ltd., Shanghai, China). LDH (A020-2-2) activity in the cell supernatant was also measured (Nanjing Jiancheng Bioengineering Institute Co., Ltd., Nanjing, China). All assays were conducted in strict compliance with the manufacturers’ instructions.

### 2.13. Histopathological Analysis

Colonic segments were fixed by immersion in 4% paraformaldehyde. They were then cleared in xylene, embedded in paraffin, and sectioned for histological examination. Tissue morphology was evaluated using hematoxylin and eosin (H&E) staining. Goblet cell numbers in the colon were measured with Alcian blue/periodic acid-Schiff (AB-PAS) staining.

### 2.14. Immunohistochemistry (IHC)

After deparaffinization with xylene and rehydration through graded ethanol, antigen retrieval was carried out in 0.01 mol/L citrate buffer (pH 6.0) at 95 °C for 20 min. Endogenous peroxidase activity was quenched by incubation with 3% hydrogen peroxide at room temperature for 10 min. The sections were then blocked with 5% BSA at 37 °C for 30 min. Primary antibodies against ZO-1 (1:200, 21773-1-AP, Wuhan Sanying, Wuhan, China) and Caspase3 (1:200, 19677-1-AP, Wuhan Sanying, China) were added, and the sections were incubated overnight at 4 °C. After rewarming, the sections were rinsed with PBS and incubated with HRP-conjugated secondary antibodies (DD13, Talent Biomedical, Xiamen, China) at 37 °C for 30 min. DAB (G1212-200T, Servicebio, Wuhan, China) was used for color development (3–5 min), followed by hematoxylin (H9627, Sigma-Aldrich, Saint Louis, MO, USA) counterstaining for 30 s. Finally, the sections were dehydrated, cleared, and mounted with neutral gum. The slides were then observed and imaged under a microscope. ImageJ 1.54p software was used for quantitative data analysis.

### 2.15. Western Blotting

Lysis of IEC-6 cell supernatants and intestinal tissues was performed in RIPA lysis buffer (R0020, Solarbio, Beijing, China) containing 1% PMSF (M5293, Abmole, Houston, TX, USA). Protein concentration determination was carried out using the BCA assay (PC0020, Solarbio, China). Protein separation by 12.5% or 7.5% SDS-PAGE (depending on molecular weight) and transfer onto PVDF membranes (Immobilon, IPVH00010, Dublin, Ireland) were then conducted. Following a 2-h blocking step with 5% non-fat milk (1172GR, BioFrox, Einhausen, Germany) at room temperature, the membranes were probed overnight at 4 °C with primary antibodies against P-AKT (1:3000, 28731-1-AP), AKT (1:7000, 10176-2-AP), PI3K (1:600, 20584-1-AP), Caspase3 (1:1200, 19677-1-AP), Occludin (1:27,000, 27260-1-AP), BCL-2 (1:2500, 26593-1-AP), and ZO-1 (1:25,000, 21773-1-AP) all from Wuhan Sanying Biotechnology Co., Ltd., Wuhan, China. The membranes were subsequently rinsed with 0.1% TBST and then exposed to HRP-conjugated secondary antibodies (1:8000, RGAR001, Proteintech, Wuhan, China) for 1 h at room temperature. Visualization of protein bands was achieved with a chemiluminescence imaging system (VILBER, Fusion-fx6 edge v.070, Collégien, France), and quantification was performed using ImageJ software. β-Actin (1:10,000, 81115-1-RR, Proteintech, China) served as the internal reference, with relative target protein expression calculated as the target-to-β-Actin ratio.

### 2.16. Statistical Analysis

Statistical analyses were performed using GraphPad Prism (version 9.5.1), with all data presented as mean ± standard deviation (SD). Data were analyzed using one-way analysis of variance and Student’s *t*-test. *A p-*value < 0.05 was considered statistically significant.

## 3. Results

### 3.1. Network Pharmacology Analysis

**Figure 2:**
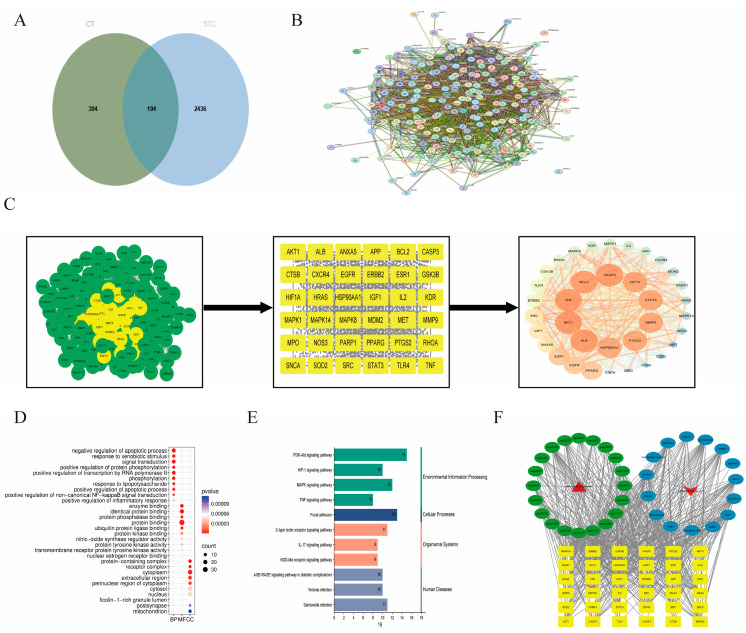
Network pharmacology analysis. (**A**) Venn diagram of potential targets of *Coreopsis tinctoria* Nutt. against slow transit constipation; (**B**) PPI network construction of *Coreopsis tinctoria* Nutt. mediated treatment of slow transit constipation targets genes; (**C**) Screening of core proteins for CT in the treatment of STC potential targets, Node size and color (blue → orange‑red) reflect the degree value (larger and darker nodes indicate higher degree); (**D**) GO enrichment analysis; (**E**) KEGG enrichment analysis; (**F**) Network analysis of herb-active component-target-disease-signaling pathway, Red upward triangles represent STC, red downward triangles represent CT, green circles represent the top 20 core pathways, blue circles represent the 15 active compounds, and yellow rectangles represent the 36 core targets.

Using network pharmacology, we retrieved and predicted 500 key targets for 15 active ingredients from various databases. The complete list of these 15 compounds is provided in Supplementary Table S1. The degree values of these active ingredients were ranked using the CytoNCA plugin in Cytoscape 3.9.1. The degree value reflects the topological importance of a node in the network, with higher values indicating more connections to target proteins. The degree value was used as the indicator for screening core compounds. The top three compounds ranked by degree value were identified as flavanomarein, rutin, and baicalin. Additionally, 2629 key disease targets associated with STC were predicted. Intersections of drug-related and disease-related targets via a Venn diagram yielded 194 common targets (Figure 2A). These common targets were imported into the STRING database to construct a PPI network and predict interactions among targets (Figure 2B). The PPI network was then analyzed in Cytoscape 3.9.1 using the CentiScape2.2 plugin for topological analysis, which screened out 36 core targets (Figure 2C). Among these, the highest-ranked targets included TNF, AKT1, Caspase3, and BCL-2.

Subsequent GO enrichment analysis indicated that these targets are predominantly involved in four core biological processes: regulation of signal transduction, regulation of apoptosis, inflammatory response, and regulation of gene expression (Figure 2D). KEGG enrichment analysis further revealed significant enrichment in key inflammatory pathways such as the TNF signaling pathway, IL-17 signaling pathway, and NOD-like receptor signaling pathway, as well as in pathways related to focal adhesion and the PI3K/AKT signaling pathway (Figure 2E). These results suggest that CT alleviates intestinal dysmotility in STC through modulating inflammatory cytokine release, improving intestinal epithelial adhesion and barrier integrity, and regulating smooth muscle cell function. Based on these findings, we constructed and visualized the target network (Figure 2F). However, given the stronger enrichment signal and more direct literature support for the PI3K/AKT pathway in inflammation and apoptosis to improve constipation, we prioritized it for subsequent experimental validation.

### 3.2. Molecular Docking

**Figure 3:**
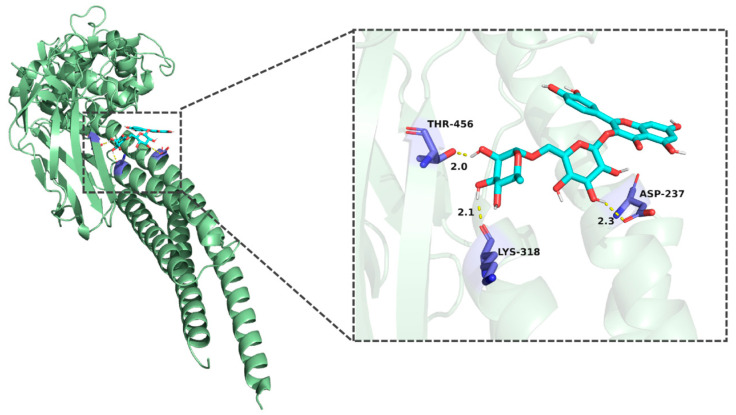
Molecular docking of rutin with STAT3.

**Figure 4:**
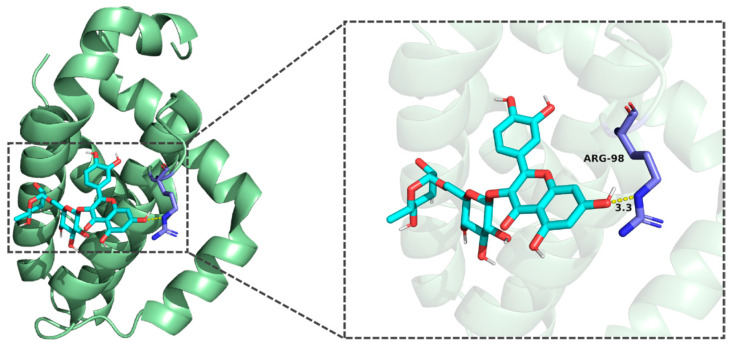
Molecular docking of rutin with BCL-2.

**Figure 5:**
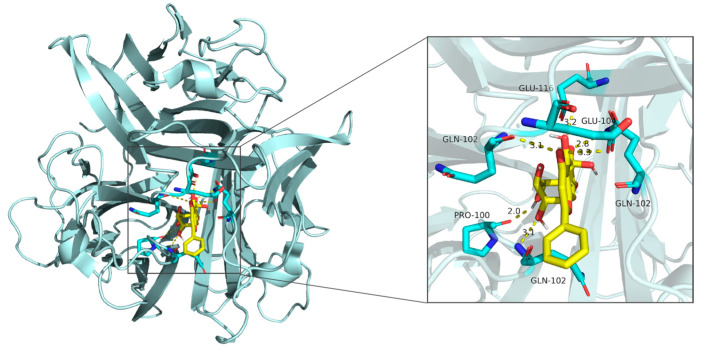
Molecular docking of baicalin with TNF-α.

**Figure 6:**
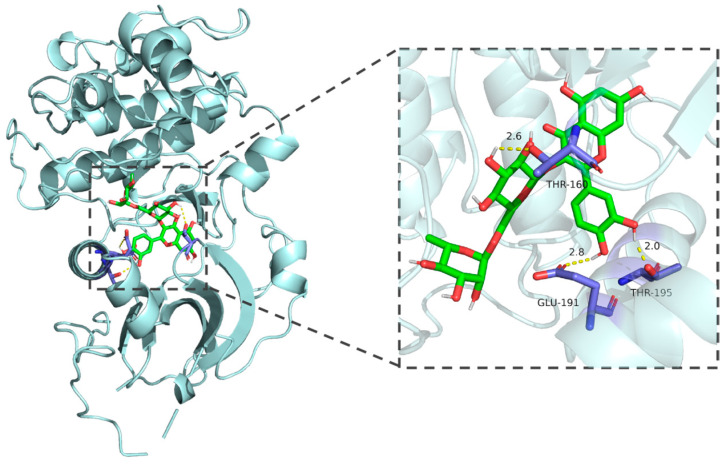
Molecular docking of rutin with AKT.

**Figure 7:**
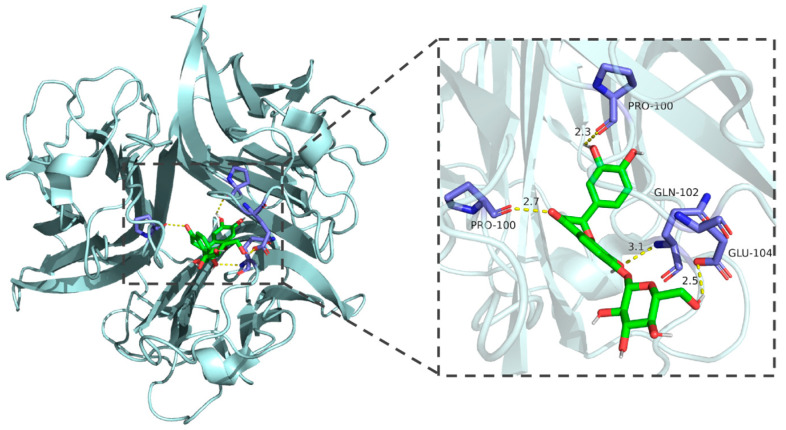
Molecular docking of flavanomarein with TNF-α.

Molecular docking results demonstrated that the binding energies of the core components of CT—flavanomarein, rutin, and baicalin—with TNF, AKT1, Caspase3, STAT3, and BCL-2 were all less than −5 kcal/mol, indicating strong binding affinity (Supplementary Table S2). The outcomes of the active molecules with lower binding energies to the target proteins were visualized using PyMOL software (Figure 3, Figure 4, Figure 5, Figure 6 and Figure 7). The RMSD values calculated by PyMOL were all below 2.0 Å, confirming the reliability of the docking protocol (Supplementary Table S2). In summary, the predictions derived from network pharmacology are reliable.

### 3.3. CT Attenuates LPS-Induced Injury in IEC-6 Cells

**Figure 8:**
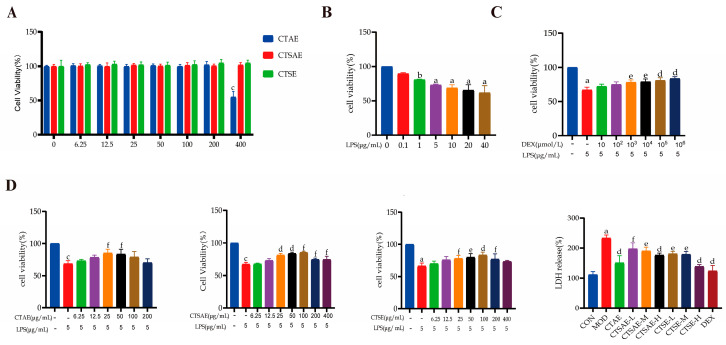
CT alleviated the LPS-induced IEC-6 cell injury model. (**A**) CCK-8 for cytotoxicity of different CT on IEC-6 cells; (**B**) Determination of the optimal LPS modeling concentration on IEC-6 cells; Impacts of DEX (**C**) and different CT extracts (**D**) on the viability of IEC-6 cells under LPS-induced injury conditions and LDH levels in different groups. Data are indicated as the mean ± SD. ^a^ *p* < 0.001 vs. con, ^b^ *p* < 0.01 vs. con, ^c^ *p* < 0.05 vs. con, ^d^ *p* < 0.001 vs. mod, ^e^ *p* < 0.01 vs. mod and ^f^ *p* < 0.05 vs. mod.

This research focuses on investigating whether CT can exert intestinal protective effects through its anti-inflammatory and intestinal barrier-repairing properties, thereby further validating the results of network pharmacology analysis. First, we conducted cytotoxicity assays on three different CT samples to determine their maximum non-toxic treatment doses. CCK-8 results showed that all three CT samples exhibited no cytotoxic effects on IEC-6 cells within the concentration range of 0–200 μg/mL (Figure 8A). After 24-h treatment with 5 μg/mL LPS, cell viability decreased to approximately 70% (Figure 8B), which was selected as the optimal modeling condition for subsequent experiments. Following LPS-induced injury, treatment with DEX significantly restored cell viability at 10^3^ μmol/L (Figure 8C), confirming this as the effective concentration for the positive control. Treatment with different CT samples at 25, 50, and 100 μg/mL significantly increased cell viability (Figure 8D); therefore, these concentrations were selected as the low, medium, and high doses for subsequent cellular experiments.

The LDH release assay (Figure 8D), a classic indicator of cell membrane integrity, the MOD group exhibited a significant increase in LDH release. Treatment with different DEX and CT samples dose-dependently reduced LDH release; the DEX group LDH release rate was significantly suppressed. The CT-SE group showed the most pronounced improvement, indicating its superior protective effect on cell membrane integrity.

### 3.4. CT Attenuates Apoptosis to Repair Intestinal Barrier

**Figure 9:**
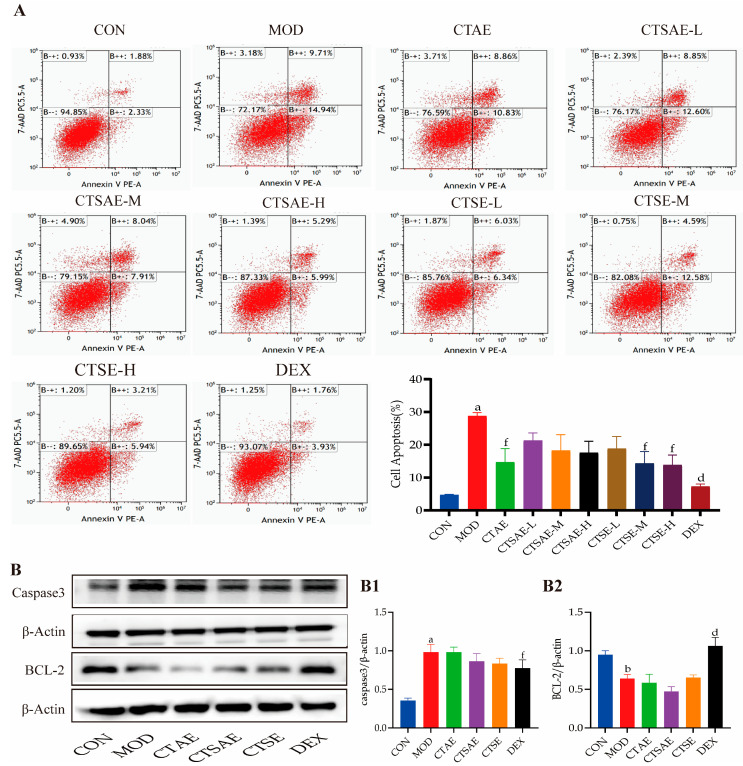
CT alleviated the LPS-induced IEC-6 cell injury model. (**A**) Apoptotic rate; (**B**) Representative blot images and the protein levels of Caspase3 (**B1**), BCL-2 (**B2**). Data are indicated as the mean ± SD. ^a^ *p* < 0.001 vs. con, ^b^ *p* < 0.01 vs. con, ^d^ *p* < 0.001 vs. mod, and ^f^ *p* < 0.05 vs. mod.

The apoptosis rate in the model group was significantly elevated, while treatment with different CT extracts and DEX, especially CTAE, CTSE-M, and CTSE-H groups, significantly reduced the apoptosis rate, with the CTSE group demonstrating more significant anti-apoptotic effects (Figure 9A). To further elucidate the molecular mechanism underlying the anti-apoptotic effect of CT extracts, we examined the expression levels of key apoptosis-related proteins, Caspase-3 and BCL-2, by Western blotting. In the MOD group, BCL-2 expression was reduced, while Caspase-3 expression was elevated. After 24-h intervention with different CT extracts and DEX, the DEX group markedly antagonized the LPS-induced changes in the aforementioned apoptotic proteins. The CTAE and CTSAE groups showed slight upregulation of BCL-2 expression and downregulation of Caspase-3 expression, but these differences were not statistically significant. In contrast, CTSE reasonably improved the abnormal protein expressions, including upregulating BCL-2 and downregulating Caspase-3 (Figure 9B). These results confirm that CTSE exerts a favorable intervention on anti-apoptosis.

### 3.5. CT Attenuates Inflammation via PI3K/AKT Pathway to Repair Intestinal Barrier

**Figure 10:**
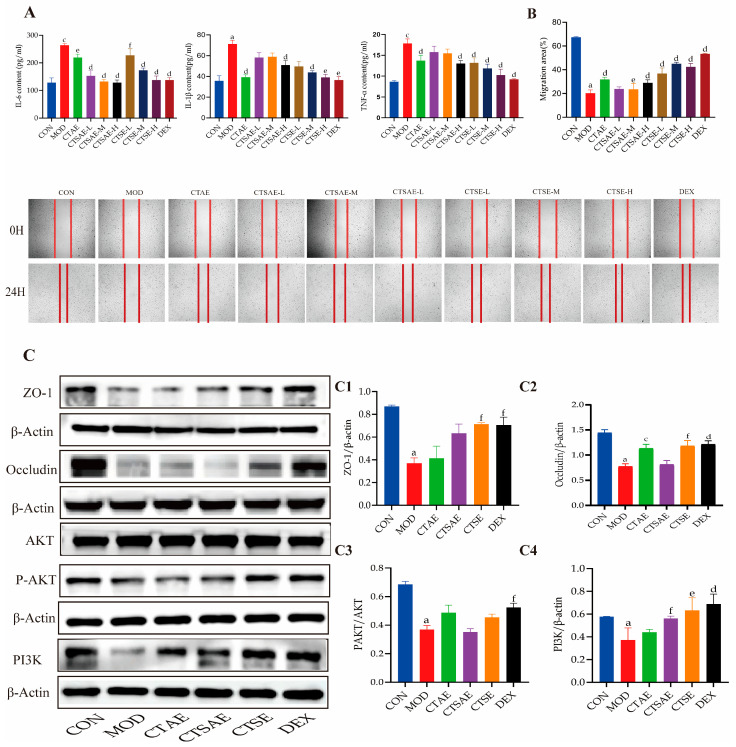
CT alleviated the LPS-induced IEC-6 cell injury model. (**A**) Levels of IL-6, IL-1β, and TNF-α in different groups; (**B**) Impact of different conditions on the cell migration; (**C**) Representative blot images and the protein levels of ZO-1 (**C1**), Occludin (**C2**), P-AKT/AKT (**C3**), and PI3K (**C4**) in LPS-induced IEC-6 cells. Data are indicated as the mean ± SD. ^a^ *p* < 0.001 vs. con, ^c^ *p* < 0.05 vs. con, ^d^ *p* < 0.001 vs. mod, ^e^ *p* < 0.01 vs. mod and ^f^ *p* < 0.05 vs. mod.

After confirming the cytoprotective effects of CT, its anti-inflammatory activity and barrier-repair potential were validated through ELISA assays and cell migration experiments, respectively. ELISA results showed that (Figure 10A), the levels of IL-6, IL-1β, and TNF-α were elevated in the supernatant of the MOD group, confirming successful LPS-induced inflammation. Intervention with the DEX group suppressed the levels of all three inflammatory cytokines, providing an effective positive reference for evaluating the anti-inflammatory effects of CT. Intervention with different CT samples led to a dose-dependent reduction in these cytokines. Both CTAE and CTSE groups at medium and high doses simultaneously downregulated all three cytokines, while low-dose CTSE only reduced TNF-α and IL-6 levels, and the CTSAE-H group selectively suppressed TNF-α and IL-6 secretion. Overall, CTSE demonstrated stable and potent anti-inflammatory activity across doses, outperforming the other samples.

The IEC-6 cell scratch wound model is widely used in studies of intestinal mucosal repair [36,37]. Scratch assay results revealed that (Figure 10B) the migration rate of the model group has decreased. This indicates that LPS injury severely impaired the repair capacity of intestinal epithelial cells. Intervention with the positive control DEX significantly promoted cell migration. Treatment with different CT samples enhanced the migration ability of injured cells, with CTSE showing the most consistent promotive effect on migration. These findings suggest that CT can accelerate intestinal epithelial repair through enhanced cell migration, supporting the restoration of the damaged intestinal barrier, with CTSE exhibiting superior reparative efficacy.

Integrated the earlier network pharmacology predictions and examined the expression of core proteins via Western blotting. In the MOD group, the expression of ZO-1, Occludin, P-AKT/AKT, and PI3K was reduced. After 24-h intervention with different CT extracts and DEX, the DEX group markedly antagonized the LPS-induced changes in the aforementioned apoptotic proteins; the CTAE and CTSAE groups showed a slight upregulation of ZO-1, but these differences were not statistically significant. CTAE and CTSE upregulated Occludin expression, while CTSAE showed a certain upward trend but no significant difference. In contrast, CTSE significantly reversed all abnormal protein expressions, including upregulating ZO-1, Occludin, P-AKT/AKT, and PI3K (Figure 10C). While all CT extracts increased P-AKT/AKT protein expression to some extent (without significant differences), CTSAE and CTSE significantly upregulated PI3K expression. These results confirm that CTSE exerts the most pronounced intervention on core regulatory PI3K/AKT pathway and consistently demonstrates optimal effects in cell protection, anti-inflammation, and barrier repair experiments.

### 3.6. Evaluation of the STC Model

**Figure 11:**
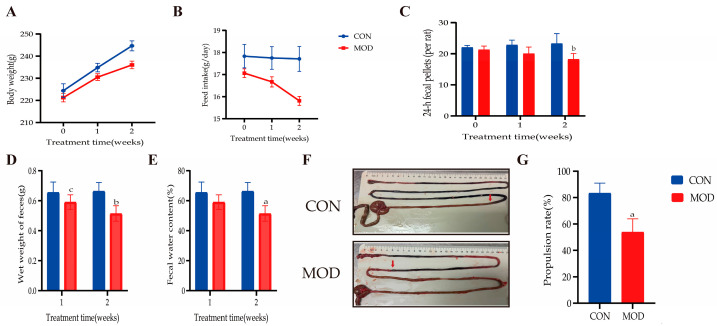
Evaluation of the LOP-induced STC model in female SD rats (days 1–14). (**A**) Body weight; (**B**) Food intake; (**C**) 24-h fecal pellets (per rat, cage-averaged); (**D**) Wet weight of feces; (**E**) Fecal water content; (**F**) Intestinal distribution of India ink (red arrows indicate the transit distance of ink); (**G**) Intestinal propulsion rate (calculated based on ink transit distance). Data are indicated as the mean ± SD. ^a ^*p* < 0.001 vs. con, ^b^ *p* < 0.01 vs. con, ^c^ *p* < 0.05 vs. con.

To confirm that the LOP-induced STC model was successfully established, we evaluated fecal characteristics and intestinal motility in female SD rats. The MOD group gained significantly less body weight than the CON group. Appetite in the MOD group was lower than that in the CON group, though this difference did not reach statistical significance. The MOD group produced fecal pellets that were fewer in number, smaller in size, harder in texture, and drier, along with delayed intestinal transit (Figure 11). These findings align with the STC model characteristics reported in earlier work [38], indicating that the STC model was successfully generated. It should be noted that, although food consumption did not differ significantly between the two groups during the modeling period, the MOD group still showed a marked reduction in fecal pellet number, which further supports the core pathophysiological feature of the STC model—impaired intestinal motility.

### 3.7. Effects of CT on Body Weight and Food Intake in STC Rats

**Figure 12:**
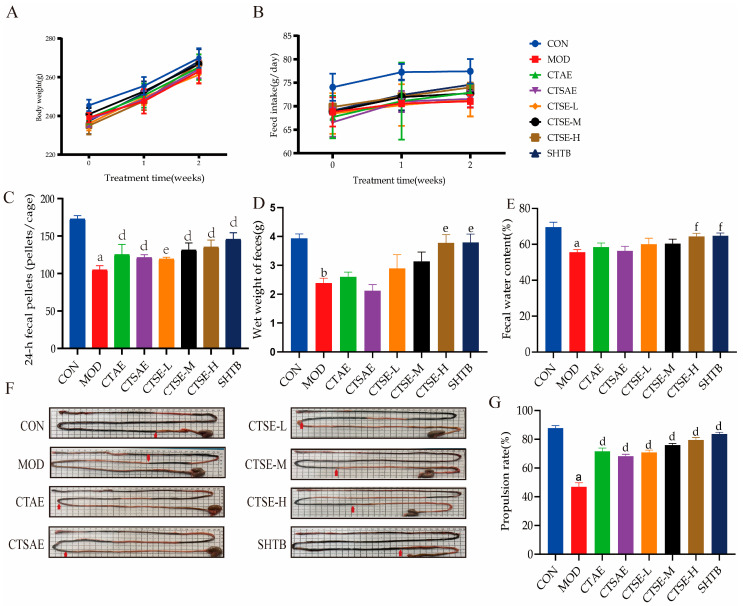
Effects of different CT samples on defecation and related indicators in LOP-induced STC model rats (days 15–28). (**A**) Body weight; (**B**) Food intake; (**C**) 24-h fecal pellets; (**D**) Wet weight of feces; (**E**) Fecal water content; (**F**) Intestinal distribution of India ink (red arrows indicate the transit distance of ink); (**G**) Intestinal propulsion rate. Data are indicated as the mean ± SD. ^a ^*p* < 0.001 vs. con, ^b^ *p* < 0.01 vs. con, ^d^ *p* < 0.001 vs. mod, ^e^ *p* < 0.01 vs. mod and ^f^ *p* < 0.05 vs. mod.

After CT intervention, body weight recovered in all treatment groups compared with the model group, with the CTSE-H and SHTB groups showing weight gain levels close to those of the CON group (Figure 12A). Furthermore, each CT treatment alleviated the reduction in food intake induced by constipation, with the CTSE-H group exhibiting the most pronounced effect. These results demonstrate that CT increases food intake and promotes weight recovery in LOP-induced constipated rats (Figure 12B).

### 3.8. Effects of CT on Defecation Ability and Small Intestinal Transit Rate

The main characteristics of STC include difficulty in defecation, reduced fecal moisture content, and weakened intestinal propulsion. All CT samples increased fecal output in STC rats and markedly raised fecal wet weight, particularly in the CTSE-H and SHTB groups. The CTAE, CTSAE, CTSE-L, and CTSE-M groups increased fecal moisture content, although not significantly, while CTSE-H and SHTB significantly enhanced fecal moisture content in STC rats (Figure 12C–E).

Studies indicate that a higher small intestinal transit rate reflects stronger intestinal peristalsis. The MOD group exhibited a significantly lower small intestinal transit rate. However, after drug intervention, all treatment groups showed a significant increase in transit rate, with the CTSE and SHTB groups demonstrating the most pronounced effects (Figure 12F,G).

### 3.9. CT Improves Intestinal Mucosal Histopathology

**Figure 13:**
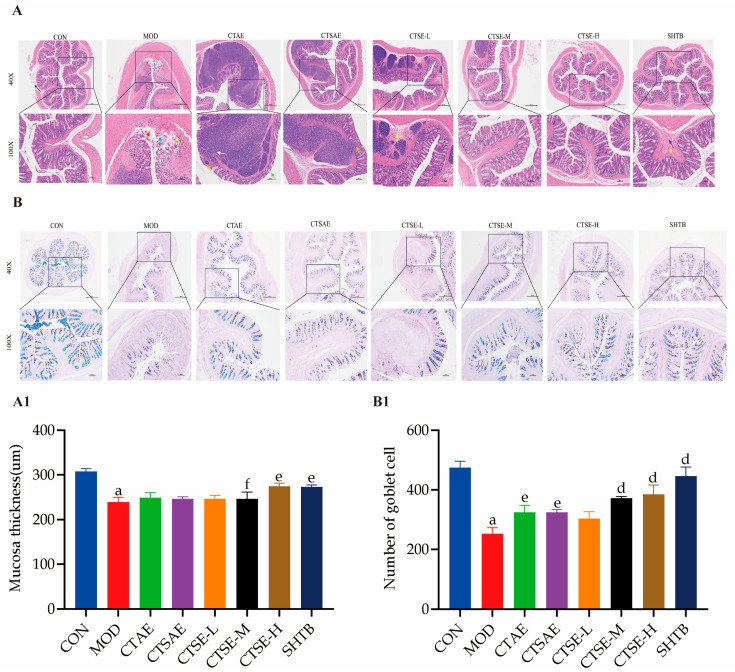
Histopathological staining results (scale bar: 500 μm/100 μm). (**A**) H&E staining; (**B**) AB-PAS staining; (**A1**,**B1**) Detection parameters; Black arrows indicate focal muscular layer hypoplasia of the intestinal tissue observed under microscopy. Red arrows indicate partial injury and disintegration of mucosal epithelial cells with diffuse structural morphology. Blue arrows indicate focal loss of intestinal gland structure with exposure of the muscularis mucosae. Yellow arrows indicate inflammatory cell infiltration. Green arrows indicate marked narrowing of the intestinal wall. White arrows indicate lymph node enlargement with involvement of the mucosal layer and discontinuity of the muscular layer arrangement. Orange arrows indicate a marked decrease in the number of intestinal glands and a significant increase in the number of lymphocytes in the mucosal layer. Purple arrows indicate connective tissue hyperplasia. Data are indicated as the mean ± SD. ^a ^*p* < 0.001 vs. con, ^d^ *p* < 0.001 vs. mod, ^e^ *p* < 0.01 vs. mod and ^f^ *p* < 0.05 vs. mod.

H&E staining results (Figure 13A) revealed severe structural damage and inflammatory infiltration in the colonic mucosa of the MOD group. After two weeks of CT intervention, the mucosal injury was notably ameliorated. Measurements of mucosal thickness indicated that the mucosa was significantly thinner in the MOD group, while the CTSE-M and CTSE-H groups’ interventions increased mucosal thickness. AB-PAS staining results (Figure 13B) demonstrated that the count of goblet cells was reduced in the MOD group. However, intervention with CT increased the goblet cell count.

### 3.10. CT Inhibits Apoptosis and Alleviates Intestinal Inflammation

**Figure 14:**
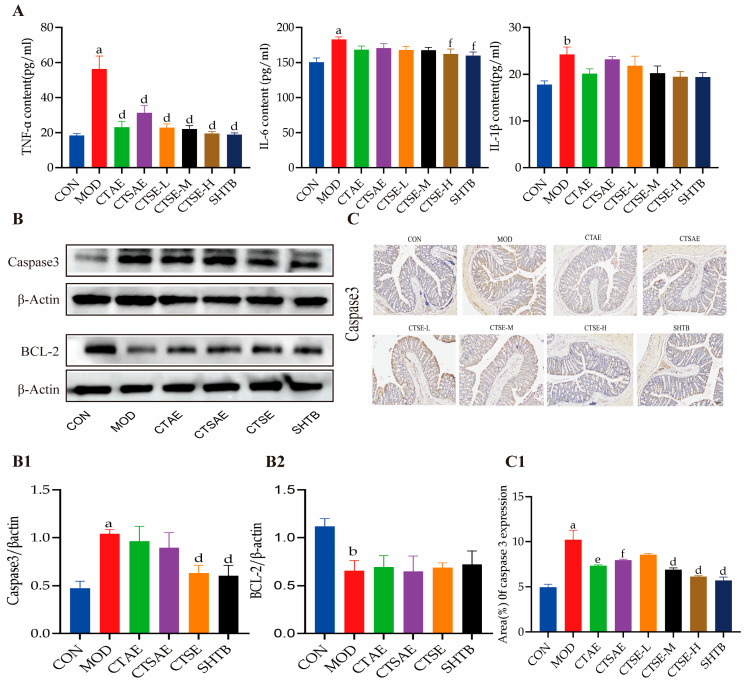
Effects of different CT samples on inhibition of apoptosis and alleviation of intestinal inflammation in LOP-induced STC. (**A**) TNF- α, IL-6, and IL-1β levels were determined in the different groups; (**B**) Representative blot images and the protein levels of Caspase3 (**B1**), BCL-2 (**B2**) in rat colon tissues; (**C**) IHC analysis of Caspase3 and the area of Caspase3 (**C1**) expression in rat colon tissues (scale bar: 100 μm). Data are indicated as the mean ± SD. ^a^ *p* < 0.001 vs. con, ^b^ *p* < 0.01 vs. con, ^d^ *p* < 0.001 vs. mod, ^e^ *p* < 0.01 vs. mod and ^f^ *p* < 0.05 vs. mod.

Intestinal barrier dysfunction may be linked to inflammatory responses and apoptosis in colonic tissue [10]. Inflammatory cytokine levels were significantly elevated in the model group (Figure 14A), indicating pronounced inflammation in constipated rats. All intervention groups reduced these inflammatory cytokines to varying degrees. The CTSAE, CTSE-L/M/H, and SHTB groups significantly lowered TNF-α levels; the CTSE-H and SHTB groups reduced IL-6 levels; and IL-1β levels in all treated groups were close to those in the CON group. These findings indicate that CT effectively suppresses abnormal release of intestinal pro-inflammatory cytokines, with CTSE showing the strongest effect.

Excessive apoptosis can disrupt barrier function [39]. Western blotting results (Figure 14B) indicated that only the SHTB group increased the BCL-2, while the CTAE and CTSE groups showed an upward trend without statistical significance. About the pro-apoptotic protein Caspase3, both the CTSE and SHTB groups reduced its expression. This result was further confirmed by IHC (Figure 14C). In summary, CT alleviates colonic inflammation and apoptosis in STC rats.

### 3.11. CT Repairs Barrier Function and Regulates the PI3K/AKT Pathway in STC Rats

**Figure 15:**
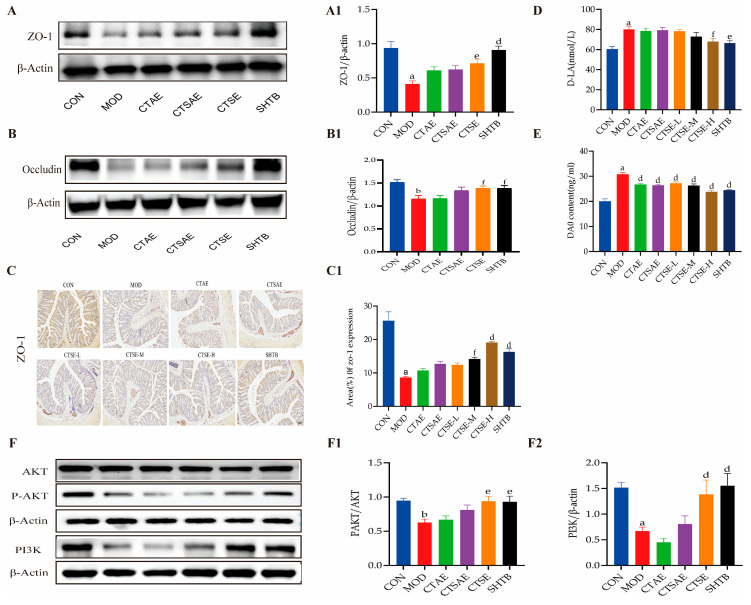
CT restores intestinal barrier function and regulates the PI3K/AKT signaling pathway in STC rats. (**A**) Representative blot images and the protein levels of ZO-1 (**A1**) in rat colon tissues; (**B**) Representative blot images and the protein levels of Occludin (**B1**) in rat colon tissues; (**C**) IHC analysis of ZO-1 and the area of ZO-1 (**C1**) expression in rat colon tissues (scale bar: 100 μm); (**D**) D-LA were determined in different groups; (**E**) DAO content were determined in different groups; (**F**) Representative blot images and the protein levels of P-AKT/AKT (**F1**) and PI3K (**F2**) in rat colon tissues. Data are indicated as the mean ± SD. ^a^ *p* < 0.001 vs. con, ^b^ *p* < 0.01 vs. con, ^d^ *p* < 0.001 vs. mod, ^e^ *p* < 0.01 vs. mod and ^f^ *p* < 0.05 vs. mod.

Constipation is linked to damage of the intestinal barrier, which is marked by a decrease in the tight junction protein ZO-1, Occludin, and an increase in permeability markers like D-LA and DAO [40,41]. Therefore, we evaluated the effects of different CT samples on ZO-1 expression and intestinal permeability in constipated rats (Figure 15A,B). ZO-1 and Occludin expression were reduced in the MOD group, indicating impaired intestinal tight junctions and barrier function. This change was reversed after CTSE treatment, a finding further confirmed by IHC (Figure 15C). In addition, the MOD group showed increased serum levels of D-LA and DAO. All CT treatment groups reduced DAO levels, while the CTSE-H and SHTB groups also showed lowered D-LA levels (Figure 15D,E).

To validate that the laxative effect of CT on rat constipation is closely associated with the PI3K/AKT signaling pathway, we investigated the expression levels of PI3K and P-AKT/AKT in the rat colon (Figure 15F). The MOD group exhibited reduced levels of PI3K and P-AKT/AKT. All CT extracts increased the expression of PI3K and P-AKT/AKT, with the CTSE and SHTB groups showing the most pronounced effects.

## 4. Discussion

Constipation is among the most prevalent gastrointestinal conditions [42]. STC constitutes the primary subtype of this disorder [43]. Major predisposing factors include poor dietary habits, psychosocial influences, environmental changes, and the accelerated pace of modern life. The rising incidence of STC, coupled with a lack of safe and effective therapeutic options, not only increases the financial burden on patients but also intensifies their psychological distress [44]. The pathogenesis of STC is complex, yet recent studies indicate that low-grade inflammation and damage to the intestinal epithelial barrier play critical roles in its development [14,45]. TCM has a long history and distinct advantages in treating constipation [46,47]. CT, a rare alpine wild plant and valued medicinal herb from Xinjiang, China, is rich in flavonoids known to modulate gastrointestinal function, suggesting its potential in addressing gastrointestinal motility disorders. However, the specific mechanisms by which CT alleviates STC remain unclear, limiting its further development. Therefore, employing network pharmacology to predict the precise mechanisms of CT in treating STC. Subsequently, we used a LOP-induced constipation rat model and an LPS-induced inflammatory injury model to simulate intestinal barrier dysfunction and validate these mechanisms. The results demonstrate that CT improves defecation-related parameters, reduces intestinal inflammation, inhibits colonic cell apoptosis, and enhances intestinal barrier function.

Network pharmacology, an emerging research methodology characterized by its network-target approach, enables systematic prediction of the regulatory mechanisms of TCM [47]. In our previous study on the total flavonoid components and intestinal absorption of CT, we screened 15 potential active compounds. By constructing an interaction network between these compounds and known STC-related targets, we identified flavanomarein, rutin, and baicalin as participants in STC regulation. We selected TNF, AKT1, Caspase3, STAT3, and BCL-2 as primary core targets. To further elucidate their biological significance, we conducted GO functional and KEGG pathway enrichment analyses. Results revealed these targets’ functions extensively involve four core biological processes: signal transduction regulation, apoptosis regulation, inflammatory response, and gene expression regulation, with significant enrichment in the PI3K/AKT signaling pathway. Therefore, we hypothesize that dichroic goldenrod may alleviate STC by modulating the intestinal inflammatory microenvironment, regulating apoptosis, and maintaining the integrity of the intestinal epithelial barrier. Based on these findings, we systematically validated this regulatory mechanism through cellular and animal experiments.

To further validate the predictions from network pharmacology, this study experimentally verified the core targets and signaling pathway by establishing an animal model of slow transit constipation induced by LOP and an LPS-induced inflammatory injury model simulating intestinal barrier dysfunction. Rats induced with loperamide exhibited typical constipation symptoms consistent with previous reports [48]. Measurements of key defecation parameters revealed that CT increased fecal water content, accelerated intestinal motility, and enhanced small intestinal propulsion rate. Histopathological examination of colonic tissue in STC rats typically demonstrated colonic muscularis thinning, inflammatory cell infiltration, reduced goblet cells, and glandular irregularities. H&E and AB-PAS staining in this study revealed extensive inflammatory cell infiltration and various pathological lesions in the distal colon tissue of the MOD group. However, treatment with different CT extracts substantially improved colonic tissue structure, with no significant inflammatory cell infiltration. Treatment significantly increased goblet cell numbers, protected intestinal barrier integrity, and mitigated LOP-induced histopathological deterioration. These findings indicate that CT improves constipation in a loperamide-induced rat model.

Building on its confirmed laxative activity, we further examined intestinal inflammation in rats, as constipation is often associated with impairments in these areas [49,50]. Patients with constipation exhibit elevated levels of pro-inflammatory cytokines TNF-α and IL-6 in their intestines [51], while dietary fiber has been shown to reduce IL-6 and IL-1β levels in constipated mice [52]. Our results indicate that intervention with different CT extracts lowered the levels of TNF-α, IL-6, and IL-1β in STC rats, with CTSE-H showing the most pronounced effect. In vitro, we treated IEC-6 cells with 5 μg/mL LPS to establish an inflammation-induced intestinal barrier dysfunction model. Model group cells exhibited decreased viability and elevated levels of pro-inflammatory cytokines, whereas intervention with different CT extracts reversed this trend. These results confirm that each CT intervention effectively counteracted inflammation-mediated cellular damage, with CTSE again demonstrating the most significant protective effect.

Apoptosis is an early event in intestinal barrier injury. In constipation, expression of Caspase3 is elevated, while expression of BCL-2 is reduced [53]. This apoptotic imbalance exacerbates barrier damage through two mechanisms: first, excessive epithelial shedding disrupts tight junctions and weakens barrier integrity; second, damage-associated molecular patterns released by apoptotic cells activate innate immune cells such as macrophages, triggering local inflammation and further increasing intestinal permeability. In this study, only CTSE significantly reduced Caspase-3 expression in colonic tissue, indicating that CTSE plays a dominant role in inhibiting apoptosis in the colonic tissue of STC rats.

Having established that CTSE effectively inhibits apoptosis, we further assessed its direct protective effects on intestinal barrier structure and function. The intestinal epithelial barrier, also called the mechanical barrier, manages the movement of substances across the intestine and is essential for host health. Prior work has linked intestinal barrier dysfunction to constipation. For instance, patients with functional constipation have lower intestinal ZO-1 expression and higher D-LA levels than healthy individuals [41]. Intestinal tight junctions primarily consist of the ZO family, the Claudin family, and Occludin, which not only mediate intercellular adhesion but also participate in regulating the transport of substances through paracellular pathways. In this study, all CT treatments increased the expression of ZO-1 and Occludin in the colon of STC rats—a finding confirmed in vitro—suggesting that CT helps maintain the structural basis of the barrier, with CTSE showing superior efficacy. In addition, Serum substances like D-LA acid, enterotoxins, and DAO can directly reflect intestinal permeability [54]. Derived from intestinal bacterial metabolism or secreted by intestinal cells as enzymes, these substances can enter the bloodstream in large amounts once the intestinal barrier is damaged. CT reduced serum levels of the intestinal permeability markers D-LA and DAO in STC rats. In vitro, CT effectively decreased LPS-induced LDH release, indicating it helps stabilize cell membranes and limit damage. Additionally, cell scratch assay results demonstrated that CT intervention significantly promoted IEC-6 cell migration following LPS injury. This indicates that CT not only protects the integrity of the barrier structure but also accelerates barrier repair by promoting epithelial cell migration, thereby further enhancing intestinal barrier function.

In the KEGG enrichment analysis, we found that the PI3K/AKT signaling pathway is a key mechanism by which CT regulates STC.TCM can alleviate constipation by activating the PI3K/AKT signaling pathway, primarily through inhibiting colonic cell apoptosis and enhancing intestinal transit [55]. Moreover, this signaling pathway is noted to play a role in regulating the function of the intestinal barrier [56,57]. Our experimental results demonstrate that PI3K protein expression levels and the P-AKT/AKT ratio were reduced in the MOD group. Following CT intervention, both PI3K expression and the P-AKT/AKT ratio increased, indicating that CT can alleviate constipation by activating the PI3K/AKT signaling pathway.

In summary, this study demonstrates that CTSE exerts the best therapeutic effect against STC, which may be attributed to the integration of two extraction technologies. Aqueous extraction mainly enriches polar flavonoids such as marein, flavanomarein, and okanin [58], among which okanin has been shown to improve intestinal barrier function [22]. Meanwhile, supercritical CO_2_ extraction efficiently enriches lipophilic components such as 2-Octyl-1-dodecanol, pentacosane, D-limonene, providing a feasible approach for retaining volatile oils and fatty oils as active substances in *Coreopsis tinctoria* Nutt. [19]. D-limonene has been demonstrated to exert gastroprotective effects and intestinal barrier repair in animal models [59], while volatile oils and fatty oils have been reported to promote intestinal motility and alleviate constipation [60]. Moreover, a study on Jichuan Decoction for STC also demonstrated that formulations containing lipophilic components exert better therapeutic effects [27]. Taken together, these results suggest that the synergy between polar and non-polar components in CTSE serves as an important basis for its superior efficacy in anti-inflammation, anti-apoptosis, and barrier repair.

This study primarily focused on the overall efficacy and mechanism of *Coreopsis tinctoria* Nutt. in treating STC, and identified the extract with better therapeutic effects. However, quantitative analysis of the main chemical components in the three extracts is still needed to determine which specific components mediate the improvement in intestinal motility. In future studies, we will compare the content of each component among the three extracts, identify the key active monomers, and further validate their efficacy and mechanisms using these monomers, with the aim of achieving more comprehensive therapeutic benefits for STC patients.

## 5. Conclusions

In summary, we have demonstrated that CT possesses significant activity against loperamide-induced STC, with CTSE treatment showing superior efficacy compared to CTSAE or CTAE treatment. CTSE is identified as the most effective strategy among the three CT extracts, and its efficacy is comparable to the positive control SHTB. This differential efficacy is likely due to the broader chemical composition of CTSE, which combines both polar (aqueous) and non-polar (supercritical CO_2_ extract) components. CT exerts its therapeutic effects by activating the PI3K/AKT signaling pathway, which mediates anti-inflammatory activity, thereby inhibiting intestinal epithelial cell apoptosis and improving intestinal barrier function. Accordingly, CT represents a promising option as a functional food or herbal medicine against STC.

## Data Availability

The original contributions presented in the study are included in the article/Supplementary Materials; further inquiries can be directed to the corresponding author.

## Supplementary Materials

The following supporting information can be downloaded at: https://www.mdpi.com/article/10.3390/cimb48050510/s1.

## Abbreviations

STC	Slow transit constipation
CT	*Coreopsis tinctoria*
ZO-1	Zonula Occludens-1
D-LA	D-lactate
DAO	Diamine oxidase
P-AKT	Phospho-protein kinase B
AKT	Protein kinase B
TCM	Traditional Chinese Medicine
LOP	loperamide
LPS	lipopolysaccharide
SHTB	Shouhui Tongbian Capsules
PPI	Protein–Protein Interaction
GO	Gene Ontology
KEGG	Kyoto Encyclopedia of Genes and Genomes
SPF	Specific pathogen-free
SD	Sprague-Dawley
IEC-6	Intestinal Epithelial Cell-6
IL-1β	Interleukin-1β
IL-6	Interleukin-6
TNF-α	Tumor Necrosis Factor-α
ELISA	Enzyme-linked immunosorbent assay
LDH	Lactate Dehydrogenase
H&E	hematoxylin and eosin
AB-PAS	Alcian blue/periodic acid-Schiff
IHC	Immunohistochemistry
PI3K	Phosphoinositide 3-kinase
