# Supplementary material for: Coreopsistinctoria Nutt. Alleviates Intestinal Barrier Damage in Slow Transit Constipation Through the PI3K/AKT Pathway
Source: Curr Issues Mol Biol. 2026 May 14;48(5):510. doi: 10.3390/cimb48050510 (PMC13204426; doi:10.3390/cimb48050510)
Supplement: Supplementary file 1 [file cimb-48-00510-s001.zip › cimb-4276986-supplementary.pdf]

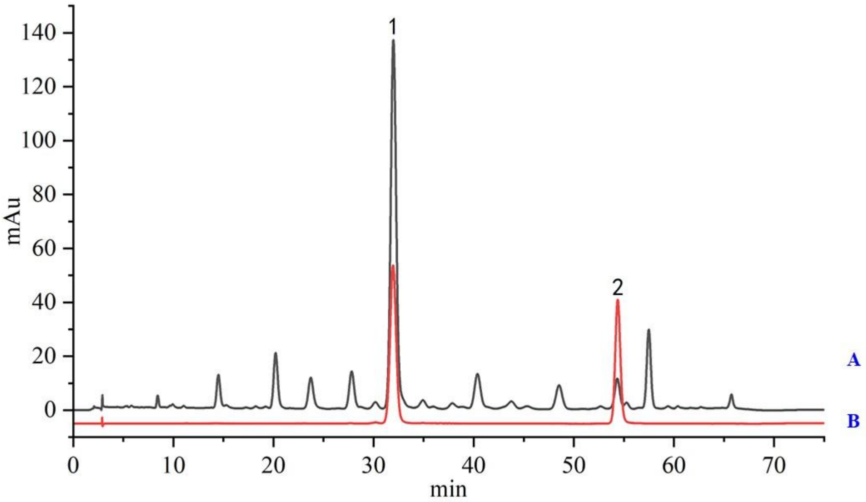

Figure S1. HPLC chromatogram of the aqueous extract of C. tinctoria (A), reference substances (B), 1: Marein；2: Okanin.

Table S1. Network Pharmacology Results of 15 Active Compounds from *Coreopsis tinctoria* Nutt.

Compound	Degree	Chemical structure
flavanomarein	131	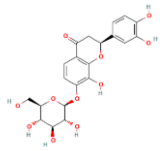
rutin	121	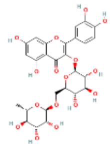
baicalin	118	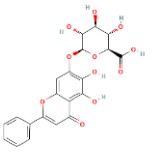
maritimein	108	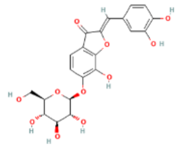
butein	107	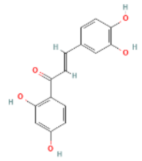

luteolin	107	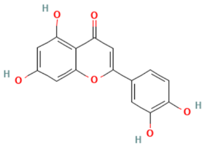
Quercetagitrin	103	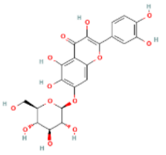
meletin	103	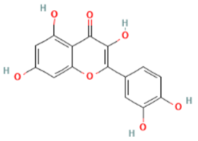
Isookanin	93	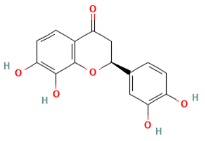
catechin	79	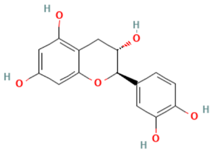
marein	44	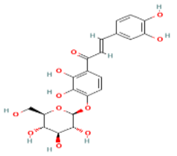
caffeic	44	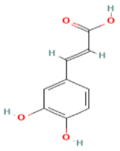
Isochlorogenic acid A	27	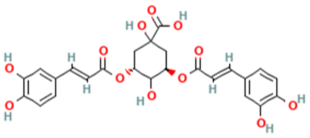
catechol	27	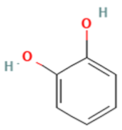
chlorogenic acid	27	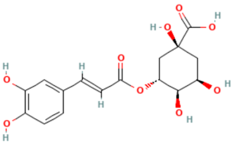

Table S2. Binding energies and RMSD values for the docking complexes of Flavanomarein, Rutin, and Baicalin with AKT, STAT3, TNF-α, Caspase-3, and BCL-2.

Compound	Hubtarget	Binding energy	RMSD

	AKT	-8.6	1.176
	STAT3	-7.8	1.181
Flavanomarein	TNF-α	-9.2	1.180
	Caspase-3	-8.2	1.183
	BCL-2	-7.2	1.182
	AKT	-9.5	0.080
	STAT3	-11.3	0.085
Rutin	TNF-α	-8.3	0.083
	Caspase-3	-8.6	0.087
	BCL-2	-10.4	0.086
	AKT	-8.9	1.166
	STAT3	-7.6	1.171
Baicalin	TNF-α	-10.2	1.169
	Caspase-3	-7.8	1.173
	BCL-2	-7.5	1.172
